# Partial prostatectomy in prostate cancer: a systematic review of current evidence

**DOI:** 10.1016/j.clinsp.2025.100777

**Published:** 2025-09-12

**Authors:** Caio Mazzonetto Teófilo de Moraes, Catharina Lyra, Alice Matos Fontes, Jose de Bessa Junior, William Carlos Nahas, Leopoldo Alves Ribeiro Filho, Caio Vinicius Suartz

**Affiliations:** a Hospital das Clínicas da Universidade de São Paulo (HCUSP), São Paulo, SP, Brazil; b Universidade Federal da Bahia (UFBA), Salvador, BA, Brazil; c Universidade Nove de Julho (UNINOVE), São Bernardo do Campo, SP, Brazil; d Universidade Estadual de Feira de Santana, Feira de Santana, BA, Brazil; e Universidade de São Paulo (USP), São Paulo, SP, Brazil; f Instituto do Câncer do Estado de São Paulo (ICESP), Universidade de São Paulo (USP), São Paulo, SP, Brazil; g Northern Ontario School of Medicine University, Ontario, Canada

**Keywords:** Systematic review, Partial prostatectomy, Prostate cancer, Prostate neoplasm, Focal therapy

## Abstract

**Objective:**

To evaluate the current evidence in the literature about partial prostatectomy as a treatment for localized prostate cancer.

**Methods:**

A systematic search of nine databases was conducted up to December 2024 to identify studies involving patients with localized prostate cancer who underwent partial prostatectomy reporting clinical epidemiological characteristics, diagnostic strategies, histopathological findings, tumor staging, functional and oncological outcomes. The primary outcome was biochemical recurrence rate. Secondary outcomes were overall survival, disease-free survival, erectile dysfunction, urinary incontinence, and perioperative complications. The extracted data were systematically synthesized.

**Results:**

The authors analyzed five studies with a total of 48 patients (three prospective and two retrospective), all with low- or intermediate-risk disease. The mean surgical duration ranged from 129.2 to 208 min, with a single Clavien-Dindo Grade III complication reported. Only one study defined and reported biochemical recurrence, with a rate of 28 %. Urinary continence was preserved in 92 %–100 % of cases, while erectile function in 40 %–100 % ‒ which may represent a point of concern for this procedure. Overall survival and disease-free survival were consistently reported at 100 %. The lack of standardized reporting and the absence of randomized clinical trials prevented the performance of a meta-analysis and hindered the applicability of the findings to clinical practice.

**Conclusion:**

Randomized clinical trials are still needed to provide stronger evidence regarding partial prostatectomy, as current data remain insufficient to support its routine use. With further research, partial prostatectomy may become an option for patients with favorable and intermediate-risk disease with unilateral lesions and concordance between magnetic resonance imaging and biopsy findings. Until then, radical prostatectomy remains the cornerstone of treatment for this patient profile.

## Highlights

• Partial prostatectomy shows promise but lacks evidence for routine clinical use.
• Partial prostatectomy has emerged as a novel surgical option aiming to preserve urinary and sexual function while maintaining oncologic control in carefully selected patients with localized prostate cancer.
• Early series suggest feasibility and safety, but long-term oncological outcomes remain uncertain compared with radical prostatectomy and focal therapies.

## Introduction

Prostate cancer is the fourth most common tumor by incidence and the eighth leading cause of cancer-related deaths worldwide.^1^ Anterior prostate cancer accounts for approximately 21 % of cases, and its location poses significant challenges for diagnosis. However, the development of multiparametric Magnetic Resonance Imaging (mpMRI), combined with guided biopsies, has enhanced diagnostic accuracy.^2^^,^^3^

The management of patients with localized prostate cancer varies by risk stratification. For low-risk disease, active surveillance is the standard of care. For intermediate-risk disease, radiotherapy or radical prostatectomy remains the standard treatment, offering the best oncological outcomes.^4^ However, in selected patients, focal therapies ‒ such as High-Intensity Focused Ultrasound (HIFU) and cryotherapy ‒ may demonstrate similar long-term outcomes and are considered at the forefront of advancements in prostate cancer treatment.^5^^,^^6^

On the other hand, the use of energy-based focal therapy in some cases may compromise the integrity of the external sphincter or the neurovascular bundles, leading to complications such as urinary incontinence, urethral stenosis, and erectile dysfunction.^7^^,^^8^ In patients with single and localized lesions in the anterior prostatic position, anterior prostatectomy ‒ targeting the regions of the gland affected by anterior prostate cancer, including the anterior fibromuscular stroma, peripheral zone, and transition zone ‒ has been proposed as an alternative to radical prostatectomy, radiotherapy, and focal therapies.^9^ Nevertheless, partial prostatectomy is not currently recommended by the major current guidelines - such as those of EAU and AUA ‒ although its use may be considered within clinical trials.^4^^,^^10^

In this systematic review, the authors aim to evaluate the current evidence regarding partial prostatectomy for the treatment of prostate cancer, focusing on its oncological, functional, and perioperative outcomes, as this analysis addresses a current gap in the literature.

## Methods

### Search strategy

This systematic review followed the Preferred Reporting Items for Systematic Review and Meta-Analysis Protocols (PRISMA-P) and was registered in the PROSPERO platform (CRD42024585805). A literature search was performed up to January 1st, 2025. The authors searched the following databases: PubMed, Embase, Scopus, Cochrane, Web of Science, Lilacs, ClinicalTrials.gov, EU Clinical Trials Register, and REBEC.

The authors utilized the following search strategy: ((("Partial prostatectomy”)) AND ((“neoplasms, prostatic”) OR (“neoplasm, prostatic”) OR (“prostatic neoplasm”) OR (“prostate neoplasms”) OR (“neoplasms, prostate”) OR (“Prostate Neoplasm”) OR (“Prostate Cancer”) OR (“Cancer, Prostate”) OR (“Cancers, Prostate”) OR (“Prostate Cancers”) OR (“Cancer of Prostate”) OR (“Cancer of the Prostate”) OR (“Prostatic Cancer”) OR (“Cancer, Prostatic”) OR (“Cancers, Prostatic”) OR (“Prostatic Cancers”) OR (“Neoplasms, Prostatic”) OR (“Neoplasm, Prostatic”) OR (“Prostatic Neoplasm”) OR (“Prostate Neoplasms”) OR (“Neoplasms, Prostate”) OR (“Prostate Neoplasm”) OR (“Prostate Cancer”) OR (“Cancer, Prostate”) OR (“Cancers, Prostate”) OR (“Prostate Cancers”) OR (“Cancer of Prostate”) OR (“Cancer of the Prostate”) OR (“Prostatic Cancer”) OR (“Cancer, Prostatic”) OR (“Cancers, Prostatic”) OR (“Prostatic Cancers”))).

### Selection criteria and screening

The authors included studies with male patients over 18-years old and prostate cancer who underwent partial prostatectomy. Our search was limited to articles in English. The authors excluded editorial letters, expert opinions, and literature reviews. Two independent authors screened all retrieved records. Discrepancies were solved with a third review. If relevant to the present review, the full text of studies cited by the screened papers was selected.

### Data extraction and analysis

The primary outcome analyzed was the biochemical recurrence rate. The secondary outcomes were postoperative erectile dysfunction, urinary incontinence, metastasis-free survival, overall survival, as well as perioperative complications, both early (up to 90-days post-operative) and late (over 90-days).

The variables considered for analysis were the number of patients, age, Charlson comorbidity index, American Society of Anesthesiologists Classification (ASA) classification, body mass index, preoperative Prostate-Specific Antigen (PSA), prostate weight and volume, prostate biopsy method, lesion volume measured with multiparametric Magnetic Resonance Imaging (mpMRI), preoperative Gleason score, International Society of Urological Pathology (ISUP) grade, D’Amico risk group stratification,^11^ prostate biopsy staging, postoperative staging, surgery duration, length of hospital stay, intraoperative blood loss, complications according to the Clavien-Dindo classification, pre and postoperative urinary continence, pre and postoperative erectile function, positive surgical margin rate, biochemical recurrence, metastasis-free survival, and overall survival.

## Results

### Literature screening

**Fig. 1:**
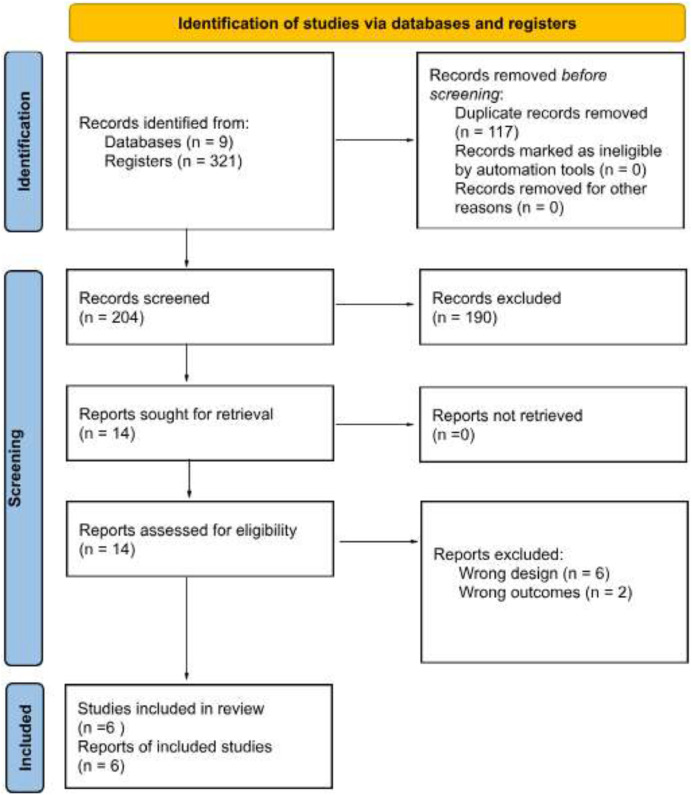
PRISMA flow chart of the selected articles.

The literature search retrieved 321 records, which were screened by title and abstract. Of these, 307 were excluded because either they were duplicates or were irrelevant to the study’s aim. The authors then reviewed the full texts of the remaining 14 studies to assess their eligibility. A total of 6 studies were excluded due to inappropriate study designs, which were cadaveric anatomic studies, editorials, and letters to the editor, and 2 studies were excluded due to wrong outcomes ‒ one study did not report outcomes from partial prostatectomy, and another had no published results at the time ‒ leaving 6 studies for inclusion in the final analysis.^9^^,^^12^, ^13^, ^14^, ^15^, ^16^ One of the publications by Villers was a continuation of the first,^9^^,^^12^ which is why the authors considered a total of five studies in this review. Fig. 1 presents the PRISMA flowchart summarizing the literature search and selection process.

### Study characteristics

A total of 48 patients were included across five studies conducted between 2000 and 2023. Four were single-arm clinical trials, one was a case series, and one was a case report. France contributed the largest share of patients (*n* = 28; 65.1 %), followed by the United States (*n* = 15; 34.9 %). Key demographic and study characteristics are summarized in Table 1.

**Table 1:** Baseline characteristics of included studies.

Study	Number of patients	Country	Study Period	Study design	Follow-up (months)	Patients’ characteristics
Villers A^9^^,^^12^	28	France	2000‒2022	Single arm clinical trial	84 (4.2 ‒ 8)	PCa at least 17-mm anterior to the rectal surface of the prostate, low or intermediate risk
Kaouk JH^13^	9	USA	2020‒2022	Retrospective Case Series	8.7 (2.4 ‒ 10.6)	Low or Intermediate risk, Gleason ≤7, organ-confined disease
Biebel MG^14^	5	USA	2022	Single arm clinical trial	9.4 (± 7.9)	Isolated anterior PCa, low-to-intermediate risk, no baseline erectile dysfunction or incontinence
Haber GP^15^	5	France	2008‒2010	Single arm clinical trial	11 (3‒15)	Isolated, anterior, low-to-intermediate risk PCa
Ferguson E^16^	1	USA	2023	Case Report	12	62-year-old male with biopsy-proven Gleason 3 + 4 PCa anterior prostate lesion

USA, United States of America; Pca, Prostate Cancer.

All patients had localized anterior prostate cancer of low or intermediate risk, confirmed by biopsy and assessed using multiparametric Magnetic Resonance Imaging (mpMRI). Surgical management consisted of robotic or laparoscopic partial prostatectomy in all cases. No study included a comparison group treated with radical prostatectomy.

### Evaluation of risk of bias

According to the ROBINS-I tool, the overall risk of bias corresponds to the domain with the highest level of bias. Accordingly, all single-arm studies were judged to have a serious risk of bias due to confounding.

Ferguson et al.^16^ did not clearly report the patient timeline or the clinical condition following the intervention. Neither the main text nor the supplementary material provided adequate details on adverse or unanticipated events. Kaouk et al.^13^ included a non-consecutive case series and lacked clarity in reporting demographic data. The studies by Villers et al.,^9^^,^^12^ Biebel et al.,^14^ and Haber et al.^15^ all showed serious confounding bias. Additionally, Villers^9^^,^^12^ and Biebel^14^ presented bias in outcome measurement. Although Haber^15^ did not exhibit this latter issue, the study demonstrated selection bias.

As a result, all five studies were deemed to have an overall serious risk of bias. Accordingly, clinical conclusions cannot appropriately be drawn from these studies, although they provide insights into partial prostatectomy. Detailed assessments are provided in the supplementary material (Supplementary Figs. 1–3).

### Baseline patient characteristics

All studies enrolled patients with low or intermediate-risk Prostate Cancer (PCa). None of them defined intermediate-risk patients as favorable or unfavorable. The Gleason score varies between 6 and 7 in all studies, and the prostate/lesion volumes also showed considerable variation across the studies. The preoperative PSA ranges from an average of 4.4 to 14.3. These characteristics are summarized in Table 2.

**Table 2:** Preoperative PSA, prostate volume, lesion volume and pre-operative Gleason score.

Study	Pre-PSA^a^ (ng/mL)	Prostate volume (cm³)	Lesion size	Gleason pre-op = *N*
Villers A^9^^,^^12^	9.6 (6 ‒ 11)	59 (42 ‒ 68)	2.3 cm³ (1.2 ‒ 4.3)	6 (3 + 3) = 13
				7 (3 + 4) = 14
				7 (4 + 3) = 1
Kaouk JH^13^	4.3 (3.7 ‒ 6.8)	32 (27 ‒ 41)	‒	6 (3 + 3) = 3
				7 (3 + 4) = 6
Biebel MG^14^	4.4 (mean)	‒	2 cm (mean)^b^	6 (3 + 3) = 1
				7 (3 + 4) = 4
Haber GP^15^	14.3 (±2.53)	‒	‒	6 or 7 (3 + 4) = 5
Ferguson E^16^	‒	‒	1.6 cm (mean)^b^	7 (3 + 4) = 1

a Pre-operative serum prostate specific antigen.

b Lesion size was given in largest diameter in this study. N, number of patients.

In the studies by Villers et al.,^9^^,^^12^ the selected patients presented a low or intermediate risk, a predominantly anterior tumor on mpMRI with at least 17 mm anterior to the rectal surface of the gland. Kaouk^13^ included patients with low or intermediate-risk disease (Gleason ≤7), organ-confined disease, and preoperative mpMRI without evidence of locally advanced or metastatic disease. Biebel^14^ included patients with isolated anterior low- to intermediate-risk PCa who underwent mpMRI with a systematic and targeted biopsy confirming an anterior mpMRI lesion. Haber^15^ included patients with an isolated, anterior, low-intermediate risk PCa who were potential candidates for targeted partial prostatectomy. Ferguson et al.^16^ described a case report of a 62-year-old male with biopsy-proven Gleason 3 + 4 prostate cancer localized in an anterior position, visible on mpMRI, as summarized in Table 1.

### Perioperative characteristics

Operative time was reported in four studies. Kaouk et al.^13^ included 9 patients, with a median operative time of 208 min (IQR: 199–211). Villers et al.^9^^,^^12^ reported 28 patients, with a mean operative time of 150 min (range: 148–188). Biebel et al.^14^ evaluated 5 patients, with a mean operative time of 129.2 min. Haber et al.^15^ and Ferguson et al.^16^ did not provide data on operative duration (Supplementary Table 1). Length of stay was described in the studies by Kaouk et al.^13^ and Ferguson et al.,^16^ and in both, all patients were discharged on the same day.

Complications other than urinary incontinence or erectile dysfunction were classified using the Clavien-Dindo system. Most patients had no complications or experienced only minor perioperative events (Grades I–II). Villers et al.^9^^,^^12^ reported Grade I–II complications in 4 patients (24 %) and a Grade III event in 1 patient (6 %). Kaouk et al.^13^ reported Grade I–II complications in 2 patients (22 %), with no Grade III–IV events. Biebel et al.^14^ observed no complications. Haber et al.^15^ and Ferguson et al.^16^ did not report data on postoperative complications.

### Functional outcomes

There was notable heterogeneity in the definition and assessment of postoperative urinary incontinence and erectile dysfunction across studies. While some studies used standardized instruments, such as the Sexual Health Inventory for Men, others relied on subjective clinical evaluation. Urinary continence was mostly preserved, with at least 92 % of patients remaining continent within three months after surgery. In contrast, erectile function outcomes were more variable, with preservation rates ranging from 40 % to 100 %. Further details are presented in Table 3.

**Table 3:** Preoperative and postoperative urinary continence and erectile function.

Study	Urinary Continence PRE	Urinary Continence POST^a^	Erectile Function PRE	Erectile Function POST^a^
Villers A^9^^,^^12^	100 %	92 %	19/28	18/19^b^
Kaouk JH^13^	100 %	100 %	23^b^	17.5^c^
Biebel MG^14^	100 %	100 %	5/5	5/5
Haber GP^15^	100 %	100 %	5/5	2/5
Ferguson E^16^	100 %	100 %	1/1	1/1

a Assessment of postoperative urinary continence varied from immediate postoperative to 3-months after surgery. Time for assessment of erectile function varied as well.

b Five of the 18 patients that maintained erectile function were dependent on phosphodiesterase inhibitors.

c Erectile function was assessed in this study using the Sexual Health Inventory for men.

### Oncological outcomes

Villers et al.^9^^,^^12^ defined biochemical recurrence as a rise in PSA levels exceeding 0.5 ng/mL/year. PSA was measured at 3- and 6-months postoperatively, and then every 6-months. In cases of progressive PSA elevation, the study recommended further evaluation with multiparametric MRI and targeted biopsies. With a median follow-up of 84-months, Villers et al.^9^^,^^12^ reported a biochemical recurrence rate of 28 %. The remaining studies did not provide a standardized definition of biochemical recurrence.

Local recurrence was assessed using serum PSA, mpMRI, biopsies, or a combination of these modalities. Kaouk et al.^13^ measured PSA at 6-weeks and then every 3-months for the first year, with transperineal biopsy and mpMRI performed at 1-year. Biebel et al.^14^ and Haber et al.^15^ measured serum PSA at unspecified intervals; Haber et al.^15^ additionally performed a multicore needle biopsy one year postoperatively. Ferguson et al.^16^ did not describe a defined protocol for recurrence surveillance.

Villers et al.^9^^,^^12^ recommended radical prostatectomy as salvage treatment for local recurrence, although most patients did not require secondary intervention. Reported survival rates without salvage treatment were 96.2 % at 1-year, 88 % at 2-years, 76.4 % at 5-years, and 62.7 % at 7- and 9-years.

Haber et al.^15^ reported one case of salvage radical prostatectomy due to a positive surgical margin in the peripheral zone, unrelated to recurrence. No other study reported local recurrence requiring salvage therapy, and none described cases of distant metastasis.

## Discussion

This study represents the first systematic review evaluating the available evidence on partial prostatectomy for patients with prostate cancer confined to the anterior region of the prostate. It is essential to emphasize that clinical conclusions cannot be drawn from these studies due to confounding bias, small sample sizes, heterogeneity in definitions ‒ such as biochemical recurrence and erectile function preservation ‒ and the absence of comparative studies. A meta-analysis could not be performed, as there was no standardization in the reporting of the studies included in this review, and their results had to be analyzed individually. With this in mind, the present analysis may serve as a foundation for insights into partial prostatectomy, upon which future randomized controlled trials may confirm or refute the findings reviewed here.

The five included studies enrolled a total of 48 patients, comprising three prospective and two retrospective studies, all involving individuals with low- or intermediate-risk disease. The mean operative time ranged from 129.2 to 208 min. Only one Clavien-Dindo Grade III complication was reported across all studies. Concerning functional outcomes, four studies reported a 100 % continence rate, while one study indicated that 92 % of patients maintained continence postoperatively. Erectile function was preserved in three studies, whereas two reported some degree of decline, with one noting a reduction to 40 % postoperative erectile function. This may represent a point of concern for this procedure. However, heterogeneity in the tools used to assess erectile function limits the ability to make direct comparisons across studies.

It is not yet possible to directly compare partial prostatectomy with focal therapies, as there are no standardized matched data, and neither approach currently has robust evidence to support consistent comparative analyses.^10^ The focal therapies described in the literature include High-Intensity Focused Ultrasound (HIFU), with or without Transurethral Resection of the Prostate (TURP), as well as irreversible electroporation, laser ablation, cryoablation, photodynamic therapy, focal brachytherapy, and radiofrequency ablation.^10^^,^^17^ Most available studies are single-arm cohorts,^10^ with a predominant focus on HIFU, occasionally compared with cryoablation.^10^^,^^17^

Cryoablation studies report local recurrence rates of up to 20 %, severe adverse events in up to 9 % of cases, near-complete urinary continence preservation, and no significant difference in erectile function compared to other focal therapies. .^10^^,^^17^ In studies evaluating HIFU combined with TURP, local recurrence rates range from 5 % to 22 %. The mean rate of severe complications (Clavien-Dindo ≥III) is 1.9 %. Urinary continence is preserved in approximately 95 % of cases, and erectile function preservation ranges from 80 % to 100 %.^10^^,^^18^ In HIFU without TURP, the median local recurrence rate is 15.4 %, and the overall complication rate is 2 %. Urinary continence is preserved in 95.5 % of patients, and erectile function preservation ranges from 88 % to 100 %.^17^, ^18^, ^19^, ^20^

Robotic-assisted radical prostatectomy yielded urinary continence rates of 83.3 %, erectile function preservation of 65.7 %, and a local recurrence rate of 8.2 %.^21^^,^^22^ In contrast, radiotherapy demonstrated urinary continence rates of 95 %, erectile function preservation of 32.8 %, and local recurrence rates at two years ranging from 7.7 % to 47.6 %, depending on radiation dose and follow-up duration.^23^^,^^24^

Current guidelines recognize focal therapy as an investigational option within research protocols but do not recommend its widespread use, given the lack of high-quality evidence directly comparing its effectiveness with radiotherapy, radical surgery, or active surveillance.^4^^,^^10^ Within this context, partial prostatectomy has emerged as an alternative for selected patients with localized tumors, although further research is still needed to consistently define its role and the patient profile that may benefit from it. Nonetheless, radical prostatectomy remains the standard of care for patients with multiple lesions, bilateral disease, posterior tumor location, or when multiparametric MRI identifies a lesion discordant with the site of the positive biopsy core.^19^

The main technical challenge reported in robotic-assisted partial prostatectomy is achieving negative posterolateral margins in the peripheral zone. In patients with larger prostate volumes, the greater amount of benign tissue between the lesion, the central zone, and the urethra increases the likelihood of negative margins. Consequently, prostate volume is considered a key factor for procedural success, with a preference for performing the surgery in glands larger than 42 mL.^9^ However, it was noted that surgeons experienced in robotic radical prostatectomy did not face significant technical difficulties when performing partial prostatectomy.^9^ Therefore, lesion size alone should not guide therapeutic decisions. Accurate preoperative planning with mpMRI is essential, and the incorporation of emerging technologies ‒ such as augmented reality ‒ into clinical practice may further improve safety, as well as functional and oncological outcomes, making this technique increasingly viable among the available procedures for the treatment of Pca.^9^^,^^19^^,^^25^^,^^26^

This study has several important limitations. The absence of randomized controlled trials and the lack of direct comparisons between partial prostatectomy and other treatment modalities limit the strength of the conclusions. It is also possible that the favorable oncological outcomes reported are, at least in part, influenced by the inclusion of lower-risk patients. Substantial heterogeneity in outcome definitions ‒ particularly regarding urinary continence and erectile function ‒ further complicates cross-study comparisons. Most patients included were from France (65.1 %), which may limit the generalizability of its findings to other regions. Moreover, none of the included studies addressed the learning curve associated with partial prostatectomy, its cost-effectiveness, or its reproducibility in routine clinical practice, which are critical for clinical adoption.

The current body of evidence is based on studies with limited methodological robustness and early-phase data. Ongoing randomized clinical trials (e.g., NCT06624813) will be crucial for determining optimal indications and for providing more definitive assessments of functional and oncological outcomes, although partial results are not yet available.^27^

Ideally, upcoming clinical trials on partial prostatectomy should standardize the reporting of outcomes, particularly oncological outcomes (e.g., local recurrence, biochemical recurrence) and functional outcomes (e.g., urinary continence and erectile function). Villers et al.^9^^,^^12^ provided a clear definition of biochemical recurrence as a rise in PSA levels exceeding 0.5 ng/mL per year, as well as a standardized protocol for assessing local recurrence using serum PSA, mpMRI, and biopsies with predefined parameters ‒ elements that could serve as a reference for future studies. Urinary continence and erectile function should be reported in accordance with international standards, such as the International Continence Society (ICS) questionnaire and the International Index of Erectile Function (IIEF), respectively.

If future studies demonstrate satisfactory oncological and functional outcomes, partial prostatectomy may emerge as a viable option for select patients with favorable or intermediate-risk disease ‒ particularly those with unilateral lesions and concordant findings between magnetic resonance imaging and biopsy ‒ where it may represent an intermediate approach: less aggressive than radical prostatectomy, yet more proactive than active surveillance.

## Conclusion

Partial prostatectomy as a focal therapy for prostate cancer is an area that still requires further investigation. Current evidence is derived from studies with limited methodological strength and short-term data. Until more robust and long-term results become available, its widespread adoption cannot be recommended.

## CRediT authorship contribution statement

**Caio Mazzonetto Teófilo de Moraes:** Conceptualization, Methodology, Validation, Investigation, Resources, Data curation, Writing – original draft, Writing – review & editing, Visualization. **Catharina Lyra:** Investigation, Resources, Data curation, Writing – original draft. **Alice Matos Fontes:** Investigation, Resources, Data curation, Writing – original draft. **Jose de Bessa Junior:** Supervision, Project administration. **William Carlos Nahas:** Supervision, Project administration. **Leopoldo Alves Ribeiro Filho:** Supervision, Project administration, Writing – review & editing. **Caio Vinicius Suartz:** Conceptualization, Methodology, Validation, Supervision, Project administration, Writing – review & editing.

## Declaration of competing interest

The authors declare no conflicts of interest.

## Appendix Supplementary materials

Image, application 1
