# Supplementary material for: Partial prostatectomy in prostate cancer: a systematic review of current evidence
Source: Clinics (Sao Paulo). 2025 Sep 12;80:100777. doi: 10.1016/j.clinsp.2025.100777 (PMC12834066; doi:10.1016/j.clinsp.2025.100777)
Supplement: Supplementary file 1 [file mmc1.docx]

**CLINICS-D-25-00461**

**Supplementary Material**

**Supplementary Table 1** Operation time and perioperative complications.

| **Article** | **Number of Patients** | **Operation duration (minutes)** | **Complications (Clavien-Dindo)^a^** |
| --- | --- | --- | --- |
| Villers A[9,12] | 28 | 150 min (148‒188) | I‒II: 4 (24%) |
|  |  |  | III: 1 (6%) |
|  |  |  | IV: 0 |
| Kaouk JH[13] | 9 | 208 min (median) | I‒II: 2 (22%) |
|  |  | IQR 199‒211 | III‒IV: 0 |
| Biebel MG[14] | 5 | 129.2 min (mean) | 0 |
| Haber GP[15] | 5 | ‒ | 0 |
| Ferguson E[16] | 1 | ‒ | 0 |

^a^ Complications according to the Clavien-Dindo classification, excluding urinary continence or erectile function.

IQR, Interquartile Range.

**Supplementary Figure 1** Quality of evidence and risk of bias in case report by Ferguson, 2023.[16]

**Supplementary Figure 2** Quality of evidence and risk of bias in case report by Kaouk, 2022.[13]

**Supplementary Figure 3** Risk of bias for all single arm clinical trials according to ROBINS-I tool.[9,11,13,14]
